# The power of consoling presence - hospice nurses’ lived experience with spiritual and existential care for the dying

**DOI:** 10.1186/1472-6955-13-25

**Published:** 2014-09-03

**Authors:** Kirsten A Tornøe, Lars J Danbolt, Kari Kvigne, Venke Sørlie

**Affiliations:** 1 Lovisenberg Diaconal University College, Oslo, Norway; 2 Norwegian School of Theology, Oslo, Norway; 3 Center for the Psychology of Religion, Innlandet Hospital Trust, Ottestad, Norway; 4 Department of Nursing and Mental Health, Hedmark University College, Elverum, Norway

**Keywords:** Dying, Spiritual and existential care, Hospice nursing, Consolation, Phenomenological hermeneutical study

## Abstract

**Background:**

Being with dying people is an integral part of nursing, yet many nurses feel unprepared to accompany people through the process of dying, reporting a lack of skills in psychosocial and spiritual care, resulting in high levels of moral distress, grief and burnout. The aim of this study is to describe the meaning of hospice nurses’ lived experience with alleviating dying patients’ spiritual and existential suffering.

**Methods:**

This is a qualitative study.

Hospice nurses were interviewed individually and asked to narrate about their experiences with giving spiritual and existential care to terminally ill hospice patients. Data analysis was conducted using phenomenological hermeneutical method.

**Results:**

The key spiritual and existential care themes identified, were sensing existential and spiritual distress, tuning inn and opening up, sensing the atmosphere in the room, being moved and touched, and consoling through silence, conversation and religious consolation.

**Conclusions:**

Consoling existential and spiritual distress is a deeply personal and relational practice. Nurses have a potential to alleviate existential and spiritual suffering through consoling presence. By connecting deeply with patients and their families, nurses have the possibility to affirm the patients’ strength and facilitate their courage to live a meaningful life and die a dignified death.

## Background

Being with dying people is an integral part of nursing, yet many nurses feel unprepared to accompany people through the process of dying [1]. Bearing witness, listening and staying present as the patients’ suffering unfolds can be emotionally challenging because it exposes the nurses to their own vulnerability and finitude [2,3]. Western society’s fast-paced healthcare environment conditions us to view death as a physiological event and a failure [4,5] rather than a natural part of the human lifecycle and a sacred passage of a life [1,6]. Easing and alleviating suffering are at the heart of nursing. As a basic category of care, the concept of suffering comprises the dying patient’s whole experience of life, health and illness in a physical, mental and spiritual sense [7].

Research indicates that a significant number of terminally ill patients experiencing spiritual and/or existential issues long for adequate spiritual or existential care and counseling [8]. Nevertheless, many healthcare professionals report a lack of skills in psychosocial and spiritual care of dying people [9-13] resulting in high levels of moral distress, grief and burnout [13]. Halifax [6] points out that we need to explore ways of being with the dying that can serve both the care giver and the dying person practically and spiritually.

There seems to be no single agreed definition of spiritual care in the nursing literature. Henceforth, this term is open to interpretation [12,14-17]. This study has adopted a pragmatic and functionalist epistemological point of departure, since it is targeted at the practical implications of the nurses’ spiritual and existential care experience, rather than the ontological questions related to the conceptual framework of spiritual care.

Nurses working with the dying and their families often witness spiritual and existential suffering [18] Hospice nurses have especially chosen to work with palliative care patients in their terminal phase. This study will therefore describe their experiences with alleviating spiritual and existential suffering, to see what can be learned about ways of being with the dying that can serve both the caregiver and the dying person practically and spiritually.

### Aim

The aim of this study is to describe the meaning of hospice nurses’ lived experience with alleviating dying patients’ spiritual and existential suffering.

## Method

### Design

The study was conducted using a qualitative phenomenological hermeneutical method influenced by Ricoeur’s philosophy [19]. This method is suitable for describing the meaning of lived experience in interview texts [20,21].

### Participants

Experienced hospice nurses from a leading hospice in Norway were invited to participate in the study. Written and oral information was given at the hospice. The first eight nurses signing up for the interview took part in the study. They were between the ages of 41 and 61 years, with eight to thirty five years of nursing experience. Everyone held advanced nursing degrees, especially relevant for end of life care, such as oncology nursing and palliative care, meeting the study’s inclusion criteria: hospice nurses with long experience in caring for terminally ill patients.

### Data collection

The interviews took place in 2012, and were conducted in the Hospice meeting room. The first and fourth author conducted the first two interviews. The first author conducted the last six interviews. The interviews took approximately one hour.

The interview strategy was designed as a narrative approach, with one open-ended question, followed by clarifying questions, when necessary.

What are your experiences with providing spiritual and existential care to terminally ill patients?

This choice was based on the underlying presupposition, that the perspective of the interviewees is best revealed in stories where the informants use their spontaneous language in the narration of events [22,23]. We did not introduce any definitions of spiritual and existential care at the commencement of the interviews, in order to allow the participants to talk about what they considered as spiritual and existential care. The aim of the interviews was to obtain as many rich narratives as possible, minimally interrupting the nurses’ narrative flow and reflection, by tying questions and comments to the informants’ responses and repeating their words whenever possible [24]. Also, the researchers were attentive to and followed up on the themes that the informants focused on in their storytelling, in order to obtain the meaning of the informants’ stories [25]. The interviews were audiotaped and transcribed verbatim.

### Data analysis

Data was analyzed using Lindseth & Nordberg’s [20] phenomenological hermeneutical method for researching lived experience, where each interview is looked upon as a text. The interpretation implies a dialectic movement between the text as a whole and parts of the text, and consists of three steps: The first step was a *naïve reading* to grasp an overall impression of the text. This gave access to the hospice nurses’ lived experience with spiritual and existential care. Keeping an open mind, the transcribed interviews were reread several times. The analysis moves towards a phenomenological world, allowing the researchers to be touched by the narratives. The naïve understanding of the text reveals the direction for the structural analysis [19,20,26,27]*The structural analysis* was the second step. The text was divided into meaning units that were condensed to themes and sub-themes. The objective of the structural analysis was to explain what the text was saying. Themes and sub-themes are presented in the Results section. As the last step, a *critical comprehension* was developed. The text was read as a whole, taking into account the authors’ preunderstanding, naive reading, structural analysis, previous research and relevant theory [20,21]. The critical comprehension is presented in the Discussion section.

### Rigour

The interview provided a large amount of in-depth information about the meaning of the hospice nurses’ lived experience. To ensure rigour, the first and fourth author performed individual structural analyses. The interpretations were compared to strengthen the credibility of the analysis. A text may have more than one possible interpretation and the interpretation presented here should be looked upon as one of several possible ways to understand the hospice nurses’ experiences [19,21]. All authors critically reviewed and discussed the interpretation of the results.

### Study limitations

This is a qualitative study. Therefore, the quest is not for objectivity and explanation (as per natural sciences) but for meanings and deeper understandings of the hospice nurses’ lived experience, and the subjective meanings of their experiences [19,28,29]. Henceforth it is not intended for inferences about incidence in the population. However the use of purposive sampling and thick descriptions may permit deeper insight into the studied phenomena and ensure sufficient information for the reader to determine whether the results are applicable to a new situation enhancing credibility [30].

### Ethical considerations

The study was conducted according to the Helsinki declaration. Approval was obtained from the Norwegian Social Science Data Service (NSD) and participants gave their written consent.

## Results

Two themes and seven subthemes from the structural analysis are shown in Table 1.

**Table 1:** Overview over themes and subthemes that emerged in the interview text

**Themes**	**Subthemes**
**Consolation**	*Consoling through Silence*
	*Consoling through Conversation*
	*Religious Consolation*
**Sensing**	*Sensing existential and spiritual distress*
	*Tuning in and opening up*
	*Sensing the atmosphere in the room*
	*Being moved and touched*

Results will be presented in the text below, including quotes to illustrate the themes.

### Consolation

#### Consoling through silence

The nurses described the patients’ emotional pain as a kind of deep existential suffering. *“Their emotional anguish can be so strong. It’s often worse than the physical pain! It’s like their hearts are being torn out! How do you relieve that kind of pain?”* The nurses experienced that sharing the silence with patients could have a powerful consoling effect. The nurses experienced that embracing the silence together with the patient, demanded a mental shift from focusing on “doing something for the patient” to focusing on “being with the patient”. This demanded personal courage. Not having a “professional mask or task” to hide behind could make them feel open and vulnerable. The nurses described how they tried to ease the suffering, by comforting and consoling through silence and active presencing: “*The only “tool” you have is yourself.”*

Communicating openness and availability was seen as an essential ingredient in spiritual and existential care. *“… just being there, sharing the pain, and letting them talk, if that’s what they need, sometimes that’s all you can do.* It was important to gauge the patients’ energy levels because they could be too tired to talk, just needing a comforting touch and a peaceful, quiet atmosphere. Silent compassion and consolation could also be communicated through a caring touch such as a gentle hand or foot massage. Sometimes silent presence and physical touch helped the patients to open up and talk about their feelings. Eventually there would come a point in time when it was too late for words. Sometimes words lost all meaning due to the brevity of the situation. *“When they are so sick that they are vomiting their own fecal matter, the only thing you can do is to be there, holding them, comforting them and warming them.”*

#### Consoling through conversation

Sometimes the nurses were able to provide a measure of consolation, by encouraging patients to talk about their situation. The nurses supported them through the emotional pain of accepting death, by listening emphatically and acknowledging their thoughts and feelings. Experience had sharpened the nurses’ courage and skills to hone in on existential and spiritual distress. As one nurse elaborated: *“People often think we have lots of conversations about life and death. Patients are usually more preoccupied with their life history and childhood memories. But being terminally ill can trigger memories about bereavement and funerals. When they bring this up I use the opportunity to ask questions like: “Have you thought about your funeral? Have you thought about your relationship with your kids? Have you thought about what’s going to happen when you pass away?* Paying attention to patients’ dreams could give access to their deeper fears. A patient told one of the nurses about a nightmare:*” I had a frightening dream. I dreamt that I disappeared…* Probing delicately to help the patient express himself the nurse asked: *“What are you anxious about? Does it have anything to do with your illness?*

It was the nurses’ impression that patients who expressed their feelings died more peacefully than those who “*bottled everything up”.* Although the nurses thought that patients usually benefitted from opening up, they respected the individuals’ choice, even if it meant clinging to a state of denial. *“Who are we to judge what is best for them?”* they reflected. The nurses strove to encourage patients to share their concerns, without invading their autonomy and integrity. As one of the nurses said: *”I try to nudge them along, encouraging them to talk about the things they long to talk about, but are afraid to express, since they don’t know how we will react.”*

The nurses emphasized that helping families to share their grief, say their goodbyes and achieve reconciliation was an important part of spiritual and existential care. They reflected on how differently families coped with the prospect of losing a loved one, and the challenges this posed. Some families shared their feelings of joy and thankfulness, as well as pain and grief, strengthening their bonds on the brink of death. Others were not able to do this. Estranged by the essential loneliness and privacy of suffering, individual family members wanted to talk with the nurse alone, turning down suggestions about having a family meeting. Trying to protect each other against the agony of death, a dying wife might say: *“I know I am dying. But I can’t tell my husband! He can’t take it”*, - or a husband could exclaim: *“I know my wife is dying. But don’t tell her! She’s too weak.”* Some times parents or children hid their emotions, putting on a “brave face” for the family’s sake. When nurses experienced that families struggled to talk, they offered to moderate the conversation, in an attempt to bridge the alienating gaps of solitary suffering.

The nurses also talked about how important it was to help patients embrace life and make the most out of their final days. Encouraging the patients’ will to live as fully as possible was also looked upon as spiritual and existential care. One of the nurses told a story that illustrated this challenge:

“There was this young woman with Cancer of the Pancreas, - her skin was all yellow and her tummy was bloated with ascites fluid. She was constantly craving Morphine and Stesolid. It seemed like she wished to float away from all her existential pain. But it was still there, underneath the drug daze. It was like a large festering boil.

I’m still not sure how we managed to puncture it, because we had tried a lot of things! But one day the doctor exclaimed: “I can understand that you must be really bitter, dying so young!” But the patient looked him squarely inn the eye, replying: ”Bitter? No I’m not bitter! This could have happened to anybody!” And the doctor started apologizing, saying: “No, No, Oh, I’m so sorry! I didn’t mean it like that! - But I thought to myself: “Yes! Exactly that question, shifted something!” because the patient started to open up: “I look at it this way: My years with this illness have made me grow and mature in ways that I couldn’t imagine, - even if I reached a ripe old age!” I remember that I responded saying: “This is so great! You really must hold on this!” And I do believe that the tears rolled down our cheeks, both on the doctor and me!

That conversation seemed to turn the situation around completely! Now we had a girl taking back her life! She went home to her apartment one last time to set her affairs in order. She took her sister and mother on a barbecue picnic, stopping at the cemetery “to take a look at where she was going to stay”. On their way “home” to the hospice, they stopped at a monument consisting of two giant lion statues. These lions stood on top of a steep flight of steps. Somehow the patient got out of her wheelchair and dragged herself up the steps. Mounting one of the lions, and striking a majestic pose, her sister took her picture. Back at the hospice she triumphantly showed me the photo, exclaiming: Isn’t it cool! I want this on the front page of my funeral program!

Reflecting on this story the nurse thought that it showed that patients could sometimes need a little push to help them embrace their lives and escape from a depressive spiral. In retrospect it seemed like the doctor had given this patient the right challenge, shaking her out of her daze. The nurses expressed that striking the right balance between challenges, mild persuasion and accepting the patients’ choices was fraught with ethical dilemmas, especially when they reflected on the patient’s vulnerability and the asymmetrical nature of the nurse – patient relationship. As they said: *“Each patient is different, and there are no easy answers.”*

#### Religious consolation

The nurses had mixed feelings about their ability to provide existential and spiritual care. The results indicate that life experiences and personal beliefs had an impact on how they felt. Talking about religious issues could generate feelings of inadequacy and insecurity. As one nurse exclaimed: *“Actually, I feel a bit uncomfortable, when patients tell me that they place their life in Gods hands. I think it’s probably because I’m not a believer. I’m very skeptical towards the Bible and the Christian faith.”* The nurses often referred the patients to the hospice chaplain for religious support. The nurses expressed a professional obligation to support the patients’ sources of faith, meaning and hope, regardless of their own personal beliefs. Talking about this obligation, the nurses reflected on their experience with accommodating the spiritual needs of Muslims. The nurses provided a prayer space, served halal food and helped patients stay in touch with their imam if this was needed. Two of the nurses had pursued theological studies prior to nursing. Colleagues regarded them as valuable resources. They helped patients with spiritual distress related to the Christian faith by combining their personal faith, theological knowledge and pastoral counseling skills. They prayed for the patients and their families and read from the Bible if they were asked to do so. They also sang religious songs with patients who found comfort in religious music.

### Sensing

#### Sensing existential and spiritual distress

The nurses described the patients’ suffering as a kind of *“total pain”*, that included emotional, spiritual and existential distress as well as physical pain. Alleviating physical symptoms was considered a prerequisite for spiritual and existential care, since unchecked pain, fatigue and nausea would drain the patients, leaving little energy for existential and spiritual concerns. The nurses experienced that spiritual and existential distress often was deeply embedded and entangled in the patients’ physical problems, making it difficult to sort out. They had therefore developed a keen eye for implicit clues, looking beyond physical symptoms. In the nurses’ experience, *“never-ending”* requests for extra pain medication or tranquilizers often suggested that the patient was having some kind of underlying emotional, spiritual or existential distress, and that this needed further looking in to. Getting a grip on what really troubled the patients could be challenging: “*I remember especially one man who was terribly restless and anxious. He couldn’t sleep. No matter how I asked him, he just said that he hurt all over. But it must have been more than physical pain because he was receiving strong analgesics through two different pumps. I often wonder if we could have done more for him. I got the impression of a very sad and lonely man”.*

#### Tuning in and opening up

Paying attention to existential and spiritual distress was important. It was also looked upon as a highly sensitive area of practice, especially if it was linked to religion.

Considered to be a deeply intrusive subject, the nurses developed various neutral and non-invasive strategies to encourage patients to express their spiritual or existential beliefs and concerns as freely as possible. At the nurses’ hospice, each patient room had a devotional calendar hanging on the wall. The calendar contained consoling scripture passages and religious hymns for each day of the year. When they sensed “the right moment”, the nurses would casually ask how the patients liked the religious calendar. Some wanted it out of their rooms, while others appreciated the calendar’s daily hymns and scripture quotes. As one of the nurses put it: *“Asking this question gives you at least a little idea of the patients’ attitude toward spirituality and religion”.* Occasionally some patients requested having the calendar quotations read to them. This would be documented in the nursing care plan: *“The patient requests daily reading from the Devotional Calendar”.*

The nurses experienced that it was challenging to pick up and interpret the patients’ signals. They placed great importance on understanding and seeing patients’ needs, and not imposing their own beliefs on them. Striving to “get it right” the nurses tried to *“tune in on”* each individual, taking time to build trust and rapport. The nurses stated that their manner of touch and tone of voice, especially during physical care, was important. *“If you do things properly, and show that you care, existential or spiritual distress eventually surfaces if it’s there.”*

In addition to implementing medical treatment and other planned activities, the nurses had to deal with acute pain, nausea and other forms of distress. Due to the complex interweaving of planned and unplanned nursing activity, and the unpredictable nature of suffering, the nurses reflected that existential and spiritual care was and had to be integrated in other aspects of care. Spending more time administering treatment due to the advances in palliative medicine, they had less time to be with patients. This created ethical dilemmas. The nurses developed strategies to find time to talk with the patients and their families: “*Some times I have to interrupt a conversation to administer a drug to another patient. It can also be difficult to walk past family members who are crying in the hallway. But I always try to indicate when I can come back and talk with them. I just hate the word “soon”. It is important to show that I care and that I have seen their distress.”* The results show that the nurses’ ability to exercise existential and spiritual care, rested on their ability to stay present, open and alert in a complex and unpredictable environment. According to the nurses, a good sense of timing, situational understanding and being able to *“tune in on”* patients’ verbal and non-verbal cues whilst performing nursing care, were necessary abilities to reach in and respond adequately to their existential and spiritual distress.

#### Sensing the atmosphere in the room

The nurses talked about how they experienced the emotional atmosphere in the patient’s room. This was especially noticeable when family members were present. A thick heavy curtain of grief blanketed some rooms: *“We feel the fear and desperation the moment we enter the room, even though it isn’t ours.* “*I have entered rooms I just have to get out of! The atmosphere is so loaded with sorrow. It’s like a physical sensation. Everything is quiet. The room is dark. The curtains are drawn. There’s no light and no energy. Nobody is talking. A whole family is just sitting there, waiting for Mom to die. The grief just hits you like a wall! How do you deal with that?*

The nurses also described rooms that were filled with joy, love and laughter in spite of the brevity of the situation. *“We never know what’s behind the door…… Sometimes patients can be quite happy! You can feel that some rooms are literally full of energy and happiness. One of our patients loved Champagne! There she sat, with family and friends, -totally bedridden, enjoying a bit of Bubbly, on the brink of death! It was just amazing!”.* A lively circle of friends and family constantly surrounded another patient. *“He often ordered take-away pizza and they had a great time together. Withdrawing gradually as his condition deteriorated, he died peacefully surrounded by his closest family.”*

#### Being moved and touched

The nurses were moved and touched by their encounters with the patients and their loved ones. Achieving rapport and building trusting relationships, easing suffering, and helping patients and their families towards peace, acceptance of dying and reconciliation, was experienced as deeply meaningful and rewarding. *“You become quite fond of the patients! Sometimes they just leap in to your heart!* Palliative care could also be emotionally demanding. Witnessing unbearable suffering and pain, in spite of all their professional and personal engagement, activated feelings of helplessness and vulnerability. The nurses placed great importance on debriefing and support from their colleagues in order to endure the emotional demands.

## Discussion

In this study the nurses narrated about their lived experiences with alleviating dying patients’ spiritual and existential suffering.

Three themes emerged through the critical comprehension, (discussion): Compassionate silence, Uncovering the wound and Wounded healers. To develop this last step in the analysis, the text was read as a whole, taking into account the authors’ preunderstanding, naive reading, structural analysis, previous research and relevant theory.

Our results show that *“being there”* for the patients and their relatives lied at the heart of the nurses’ spiritual and existential care practice. *“Being there “* was about conveying consolation through silent presencing, companionship, deep existential and religious conversations, and by supporting the patients’ expressions of faith and rituals. The results suggest that the nurses were able to use silence in a therapeutic and consoling manner, alternating skillfully between i*nvitational* and *compassionate silence.* Through caring presence and shared silence, the nurses offered a context and space where patients might feel safe enough to open up, and express their existential, spiritual or religious concerns, allowing the nurses to help them interpret their suffering in a meaningful way.

Over several years the nurses’ mental focus had shifted from “doing something” for the patient” to “being with the patient”. They had learned through experience that modern medicines’ emphasis on “doing”, “fixing” and “curing” needed to be balanced with the quality of being present *with* the dying and their families [13]. According to Rushton et al [13], presence refers to the capacity to be fully there with a quality of attention and authenticity that informs relationships and actions. When nothing else can be done, bearing witness to suffering are healing acts in themselves and are often “enough” [13]. Covington [31] points out that caring presence has been discussed in the nursing literature as a way that nurses can be with patients to provide an atmosphere of shared humanness and connection.

This resonates well with our results. In the following, the nurses’ use of silence will be discussed in light of Back et al’s [32] research. According to them silence may increase the nurses’ awareness of patients’ facial micro expressions or barely perceptible changes in voice tone, thus helping them to “tune in on” existential and spiritual distress. However, Back et al [32] point out that consoling through silence is not simply a matter of withholding speech. Drawing on several studies, they discuss how silences are filled with texture and feeling, and may have therapeutic, neutral or destructive effects on the therapeutic relationship. Silences can feel awkward, indifferent or even hostile, or comforting, affirming, and safe. If the clinician looks uncomfortable, generating palpable feelings of unease, this may be transmitted and misinterpreted by patients as i.e. judgment, disapproval, and ambivalence. Opposed to awkward silences, comforting silences resonate with the ease of patients and nurses exchanging feelings and thoughts that do not quiet make it into language. Back et al [32] comment on communication teachers’ frequent recommendations of using *invitational silence.* Here silence is deliberately created to invite patients to think, feel and express themselves. While acknowledging the value of invitational silence; Back et al [32] advocate a form of *compassionate silence* that has received little attention in health care. According to them, *compassionate silence is* a kind of silence that emerges spontaneously in the conversation, often when the clinician and patient share a feeling, or the clinician is actively generating a sense of compassion for the patient. *Compassionate silence* can be experience as a profound kind of “being with” and “standing with” in a difficult moment. It can nurture a mutual sense of understanding and care, and may be a means to console and ease the loneliness of suffering. However, our results also reveal that sometimes, just sharing the silence is not enough. In the nurse’s story about the young woman with Cancer Pancreas the nurse spoke of the need to *“puncture the boil”* in order to reach in to the patient’s existential and spiritual suffering.

In the following this will be discussed in light of Norberg et al’s [33] study of the phenomenon of consolation. According to them, *“uncovering the wound”, (the cause of the suffering)* is a necessary albeit painful part of the consolation process. In the short run, uncovering the wound (or puncturing the boil) increases the patient’s pain, because the wound becomes obvious, uncovering all that is ragged and broken. However, in the long run, exposing the wound will alleviate the pain and loneliness of suffering. According to Norberg et al [33] both parties must become ready for consolation before they are able to mediate or receive it. This involves becoming open, present and available. The nurse mediating consolation becomes “ready” through a willingness to see the wound and listen to the suffering person.

Our results show that becoming willing and ready to “just be there”, seeing and listening to the wounded patient demanded personal courage, especially when they were caring for desolate and despairing patients. Facing patients’ suffering could sometimes open the nurses’ own wounds and trigger feelings of helplessness, vulnerability, and uncertainty. The nurses expressed that giving and receiving peer support and debriefing was vital to endure the emotional pressures of being with the dying. This is in line with Miller et al’s [34] research. They point out that nurses must learn to embrace and explore their own wounds, by focusing on their own pain, and reappraising it as a source of energy and growth, literally becoming “*wounded healers”*. This will place them in a more informed position to understand when they will be able to provide compassion and when they may be harmful to others [34].

In Norberg et al’s [33] consolation study, the nurse mediating consolation “walks alongside” accepting the patient’s expressions of weakness, grief and pain. The suffering patients become ready for consolation by expressing their feelings. The nurse mediating consolation and the suffering patient become able to “see” and confide in each other: *” A trusting relationship creates room to uncover the wound and look at the cause of suffering. The suffering person becomes calmer and dares to look at the wound”*[33]. According to them, when the wound is uncovered, in communion and dialogue, a shift of perspective takes place, enabling the suffering person to become free of the overwhelming feelings of darkness and petrification so that he or she is able to contemplate his or her suffering and reach a feeling of how to relate to it. This enables the person to place suffering within a pattern of meaning. Receiving consolation generates a shift of center for the suffering person, enabling her to “break through her shell”, and undergo new experiences and through them renew and enhance her life.

In the nurse’s narrative, the doctor’s uncensored outburst about the young woman’s “*presumed bitterness”* can be interpreted as literally uncovering her wound, thus, liberating her to express her thoughts and feelings. This seemed to create a turning point in the patient’s life by snapping her out of her drug daze. The patient rose from her deathbed, reconnecting with her will to live and her family. By visiting the cemetery with her sister and mother, and mounting the lion statue, the patient broke through her shell, transcended her suffering and came to terms with her impending death, regaining communion and unity with her family.

When the nurses were asked to narrate about their experiences with existential and spiritual care, their story themes evolved around sensing the patients’ existential and spiritual distress, and their attempts to mediate consolation. Whether the nurses managed to convey consolation or not varied. However, the stories reveal that the nurses’ consolation efforts were aimed at assisting the patients towards a good death. In their stories, “consoled” patients “died well”. Expressing awareness, acceptance and preparation for death, these patients died in a peaceful and dignified manner. The “un-consoled” patients were characterized by a lack of acceptance of death and a failure to actively pursue fulfillment of living in the final scenes of dying. These “un-consoled patients” were looked upon as problematic and emotionally challenging, forming the focus of peer support and debriefing. This is consistent with the research of Hart et al [35]. The hospice movements’ “good death ideology” could be recognized in the nurses’ attitudes about dying. This ideology involves an open awareness of dying, open communication, a gradual acceptance of death and settling of both practical and “interpersonal business”. Just as members of contemporary society are expected to age well with the aide of technology and a youthful spirit, dying people are expected to live well until they die and make their own choices in the process [35,36]. Taking this into consideration, the nurses’ story about the young woman can be interpreted as a story about a “good hospice death”.

However, the concept of “the good hospice death” is critiqued and debated in the literature. Hart et al [35] discuss the underlying ideology of the “good death concept” as a means for social control, stating that the choices and opportunities of dying people are powerfully shaped and constrained by those caring for them, and that patients may implicitly be expected to conform to the normative values of the ”good death concept ”. This can be ethically problematic. As McNamara [36] points out, patients may not necessarily comply with a “good death model” that involves awareness, communication and acceptance. An array of factors including the patients’ physical and mental capacity, and their right to chose or refuse certain treatments and therapies influence the patients’ choices about how they wish to live and die. The results show that the nurses were aware of these ethical dilemmas, as they reflected on how they could encourage patients to express their distress without violating their dignity and autonomy.

Although the nurses thought that patients usually benefitted from opening up, they respected the individuals’ choice, even if it meant clinging to a state of denial. *“Who are we to judge what is best for them?”*

## Conclusions

Consoling existential and spiritual distress is a deeply personal and relational practice, involving a high degree of sensitivity and the courage to *just be there* as wounded healers, willing and ready to share the fear of death and the finitude of life with patients and their loved ones. The results suggest that through the power of consoling presence, nurses have a potential to alleviate existential and spiritual suffering. By connecting deeply with patients and their families, nurses have the possibility to affirm the patients’ strength and facilitate their courage to live a meaningful life and die a dignified death.

## Competing interests

The authors declare that they have no competing interests.

## Authors’ contributions

KT, VS, and LJD designed the study. KT and VS collected the data and performed the structural analysis. KT drafted the manuscript. KT, VS, LJD, KK contributed to the interpretation of the results, and critical review of the manuscript. All authors read and approved the final manuscript.

## Authors’ information

KT. PhD. student, Norwegian School of Theology and Center for the Psychology of Religion, Innlandet Hospital Trust Norway, RNT, Associate professor Lovisenberg Diaconal University College, Norway.

LJD. Professor Dr. Theol., Norwegian School of Theology, Director of The Center for the Psychology of Religion, Innlandet Hospital Trust, Norway.

KK. Professor, PhD., RNT. Department of Nursing and Mental Health, Hedmark University College Norway.

VS. Professor, PhD., RNT. Lovisenberg Diaconal University College, Norway.

## Pre-publication history

The pre-publication history for this paper can be accessed here:

http://www.biomedcentral.com/1472-6955/13/25/prepub
